# Fronto-parietal theta high-definition transcranial alternating current stimulation may modulate working memory under postural control conditions in young healthy adults

**DOI:** 10.3389/fnhum.2023.1265600

**Published:** 2023-11-06

**Authors:** Yanwen Xiao, Junhong Zhou, Rong Zhou, Yu Liu, Jiaojiao Lü, Lingyan Huang

**Affiliations:** ^1^Key Laboratory of Exercise and Health Sciences of Ministry of Education, Shanghai University of Sport, Shanghai, China; ^2^Department of Rehabilitation Medicine, Shenshan Medical Center, Sun Yat-sen Memorial Hospital, Sun Yat-sen University, Shanwei, Guangdong, China; ^3^Hinda and Arthur Marcus Institute for Aging Research, Harvard Medical School, Boston, MA, United States

**Keywords:** HD-tACS, working memory, postural control, dual-task, resting-state EEG

## Abstract

**Objects:**

This study aimed to investigate the immediate effects of fronto-parietal θ HD-tACS on a dual task of working memory-postural control.

**Methods:**

In this within-subject cross-over pilot study, we assessed the effects of 20 min of 6 Hz-tACS targeting both the left dorsolateral prefrontal cortex (lDLPFC) and posterior parietal cortex (PPC) in 20 healthy adults (age: 21.6 ± 1.3 years). During each session, single- and dual-task behavioral tests (working memory single-task, static tandem standing, and a dual-task of working memory-postural control) and closed-eye resting-state EEG were assessed before and immediately after stimulation.

**Results:**

Within the tACS group, we found a 5.3% significant decrease in working memory response time under the dual-task following tACS (*t* = −3.157, *p* = 0.005, Cohen’s *d* = 0.742); phase synchronization analysis revealed a significant increase in the phase locking value (PLV) of θ band between F3 and P3 after tACS (*p* = 0.010, Cohen’s *d* = 0.637). Correlation analyses revealed a significant correlation between increased rs-EEG θ power in the F3 and P3 channels and faster reaction time (*r* = −0.515, *p* = 0.02; *r* = −0.483, *p* = 0.031, respectively) in the dual-task working memory task after tACS. However, no differences were observed on either upright postural control performance or rs-EEG results (*p*-values <0.05).

**Conclusion:**

Fronto-parietal θ HD-tACS has the potential of being a neuromodulatory tool for improving working memory performance in dual-task situations, but its effect on the modulation of concurrently performed postural control tasks requires further investigation.

## 1. Introduction

Working memory (WM) is a complex capacity-limited system responsible for simultaneously maintaining an active state of being (Baddeley, 2012). The WM model presented by Baddeley and Hitch (1974) assumes a central executive component that allocates cognitive resources according to task demands, enabling people to perform a wide range of complex cognitive activities simultaneously as well as multitasking (Baddeley and Hitch, 1974; Conway et al., 2008; Baddeley, 2012). Previous studies have found that WM is a core executive function that supports dual-task locomotor performance in childhood and adolescence, and decreased WM is associated with poorer dual-task walking and upright standing in older people (Montero-Odasso et al., 2009; Hocking et al., 2020). Behavioral studies have compared dual-task performance on different types of cognitive tasks (Bernard-Demanze et al., 2009; Chen et al., 2018). They found that working memory may interfere with postural stability. This may be because postural control competes with the WM task for limited attentional resources. Furthermore, neuroscientific techniques have provided evidence in favor of a relationship between WM and postural control. Electrophysiological evidence indicates that the dorsolateral prefrontal cortex (DLPFC) and posterior parietal cortex (PPC) may provide the neural underpinnings for working memory processes (Curtis and D’Esposito, 2003; Wang et al., 2018). The frontal and parietal cortices are thought to be associated with postural control. An fNIRS-based study found that the bilateral dorsolateral PFC and frontal visual fields play important roles in maintaining standing balance (Mihara et al., 2008). An event-related potential study found a postural control-evoked N1 component in motor cortex regions (Little and Woollacott, 2015). Ozdemir et al. (2016) found that under a working memory-postural control dual task, δ, θ, and γ oscillations were increased and predominantly on the frontal, central frontal, central, and parietal cortex.

Collectively, these studies suggest that postural control and WM processes share similar neural control bases. Furthermore, cognitive training (e.g., WM) have been shown to improve both motor tasks and dual-task postural control (Borel and Alescio-Lautier, 2014; Kimura et al., 2017). Therefore, enhancing cognitive-motor dual-tasking ability by improving WM function may be feasible.

Transcranial alternating current stimulation (tACS), a type of transcranial electrical stimulation (tES), is a method of driving intrinsic cortical oscillations in electrically stimulated target areas with sinusoidal alternating current, predetermined frequency, and phase parameters (Zaehle et al., 2010; Antal and Paulus, 2013). Increasing evidence indicates that frontoparietal theta tACS has great potential for improving WM, which depends on the neurophysiological mechanisms of working memory (Polanía et al., 2012; Violante et al., 2017). Electrophysiological evidence has indicated that θ oscillatory mechanisms (phase synchronization of θ oscillations) in the fronto-parietal cortex play an important role in working memory (Wu et al., 2007; Kawasaki et al., 2010; Sauseng et al., 2010; Xu, 2017). Recent advancements in tES technology have rendered it, it possible to target brain areas more focally using high-definition tACS (HD-tACS) (Reinhart and Nguyen, 2019; Klírová et al., 2021). HD-tACS can elicit more focal stimulation to increase the confidence of spatial inference by surrounding stimulation electrodes with oppositely polarized return electrodes. To more precisely modulate the fronto-parietal region, HD-tACS was used as the modulation tool in this study.

However, it is unknown whether fronto-parietal HD-tACS induced modulation of cortical oscillations within these regions can enhance the ability to stand while simultaneously performing a working memory task. Therefore, we designed a dual-task paradigm of working memory-postural control, using fronto-parietal HD-tACS at 6 Hz as the intervention, and hypothesized that (1) fronto-parietal HD-tACS at 6hz can improve postural control as well as working memory capacity. In addition, to analyze the neuromodulatory mechanisms of HD-tACS, we recorded resting-state EEG (rs-EEG) before and after the intervention and we assumed that (2) EEG θ power and phase synchronization of θ band would be enhanced upon active tACS compared to sham; the electrophysiological effect would be in line with behavioral effects.

## 2. Method

### 2.1. Participants

For this experiment, a group of 20 healthy young adults, comprising 8 males and 12 females, were carefully selected. All participants were thoroughly informed about the risks and requirements of the experiment, and they willingly agreed to participate by signing an informed consent form. The experimental protocol was reviewed and approved by the Ethics Committee of Shanghai University of Sports. Furthermore, all the participants had right-sided dominant legs, which was determined by asking them to kick a ball (van Melick et al., 2017). Exclusion criteria included: (1) any acute illness requiring hospitalization within the past 3 months; (2) the use of neuroactive drugs that affect the brain state; (3) any self-reported cardiovascular or cerebral diseases, neurological diseases, musculoskeletal disease, or any other disease that could affect upright standing; and (4) any contraindications concerning the use of tACS (e.g., mental-implanted devices in the brain). The basic profiles of the participants are presented in Table 1.

**Table 1:** Basic profile of participants (mean ± SD).

Number	Age (year)	Height (cm)	Weight (kg)	Edu. years
20	21.6 ± 1.3	169.1 ± 9.2	65.7 ± 13.5	15.8 ± 1.7

### 2.2. Procedure

A double-blind, randomized, sham-controlled within-subjects crossover was completed, wherein participants were randomized to receive active or sham tACS: (1) fronto-parietal tACS at 6hz; (2) sham tACS (sham) with a minimum interval of 72 h between each session. Participants were required to visit 3 times. During the first visit (visit 1), also known as the screening visit, basic demographic information was collected, including age, sex, weight, height, ethnicity and living status. Participants also completed a cognitive test consisting of six blocks to determine their cognitive level of difficulty. The test had to be completed with an accuracy level of 0.6–0.8, and this level would be used in the subsequent visits. Of note, three participants had to be excluded from the study during visit 1 as their accuracy was deemed too high, which could have affected the results. Finally, of the 20 participants who were formally included in the study, the working memory test had an accuracy of 0.73 ± 0.06 as measured during visit 1. (2) Visits 2–3: all participants first performed a brief familiarization of the task. Single-task (cognitive single task × 3 or postural control single task × 2) and dual-task (cognitive task + postural control) × 2 were assessed before and after tACS. We numbered 20 participants and created a randomized list to counterbalance the order of assessments across participants. Each task lasted for approximately 90 s.

**Figure 1:**
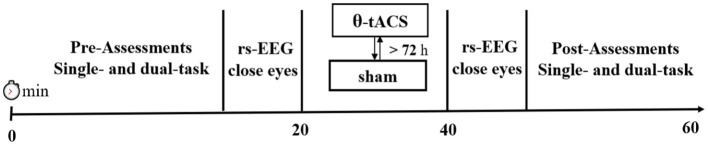
Overview of the experimental protocol.

Additionally, we recorded resting-state EEG with eyes closed for 3 min before and after the stimulation intervention to investigate the effect of tACS on endogenous brain oscillations (Figure 1).

### 2.3. Assessments

#### 2.3.1. Working memory task

**Figure 2:**
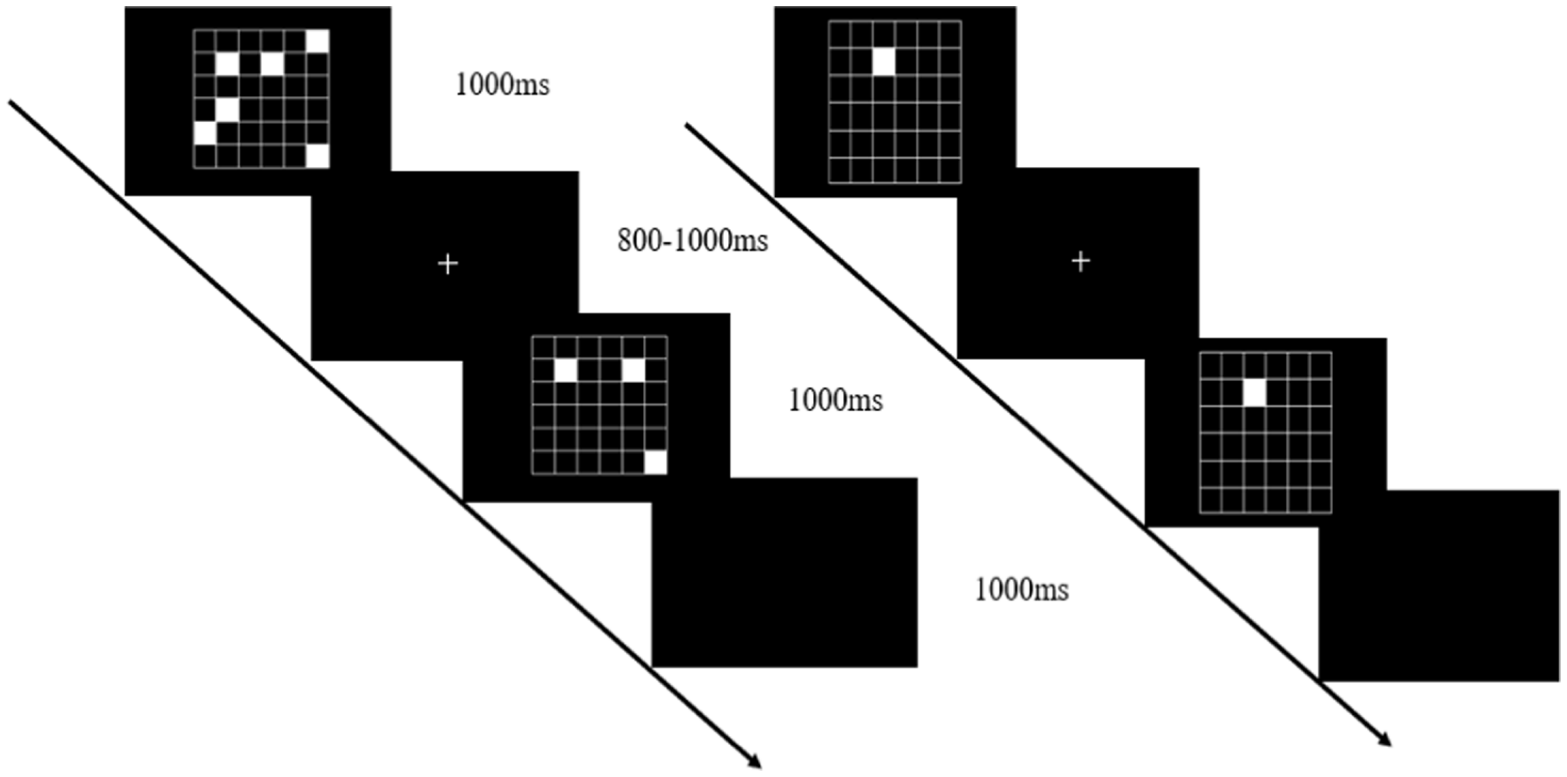
Working memory task: the visual-spatial match-to-sample test (left), and the cognitive control task (right).

The working memory task was presented on a computer screen (1920 × 1,080 pixel resolution, 60 Hz refresh rate) 1 m from the participant’s eyes. Stimulus presentation was controlled using Matlab’s Psychtoolbox software package. Working memory was tested using a visual-spatial match-to-sample task (Figure 2). The cognitive tasks were presented at different levels of difficulty and varied by manipulating the number of target stimuli to be contrasted with a subsequent probe. In this task, there were 4 events in each trial, including a target stimulus for 1,000 ms (a set of 4–8 randomly scattered white squares appearing on the screen), a memory maintenance period of 800–1,000 ms, a probe image for 1,000 ms (2–4 white squares appearing on the screen), and a response judgment period of 1,000 ms. The participants had to determine whether the latter white squares were in the same position as any previously displayed white squares. If they match, press the left mouse button; otherwise, press the right mouse button. There were 20 trials in each block with 50% of the target stimuli. During the working memory single task, participants completed the three blocks test while in a seated position, and during the dual task, they were asked to complete the working memory test while standing.

Working memory outcomes included the accuracy (ACC) of the working memory task, reaction time of accurate responses (RT), and inverse efficiency score (IES). The IES represents the ratio of response time to accuracy during cognitive task completion, and can be interpreted as the average energy consumed by the system during the trial (Townsend and Ashby, 1983; Bruyer and Brysbaert, 2011), and is calculated as follows

$${IES}_{i,j}=\frac{\bar{{RT}_{ij}}}{{PC}_{ij}}$$

where RT_*i*,*j*_ is participant *i*’s mean RT on correct-response trials in condition *j* and PC_*i*,*j*_ is participant *i*’s proportion of correct responses in condition *j*. Note, PC here has the same meaning as accuracy (ACC) in this article.

#### 2.3.2. Standing postural control

The performance of standing posture control was measured and recorded using Super Balance (ACMEWAY, Beijing, China) at 100 Hz. The participants maintained a static tandem stance (choosing the right leg as the front leg) for 60 s × 2 during the execution of the single and dual tasks. There was no task prioritization during the assessments.

**Figure 3:**
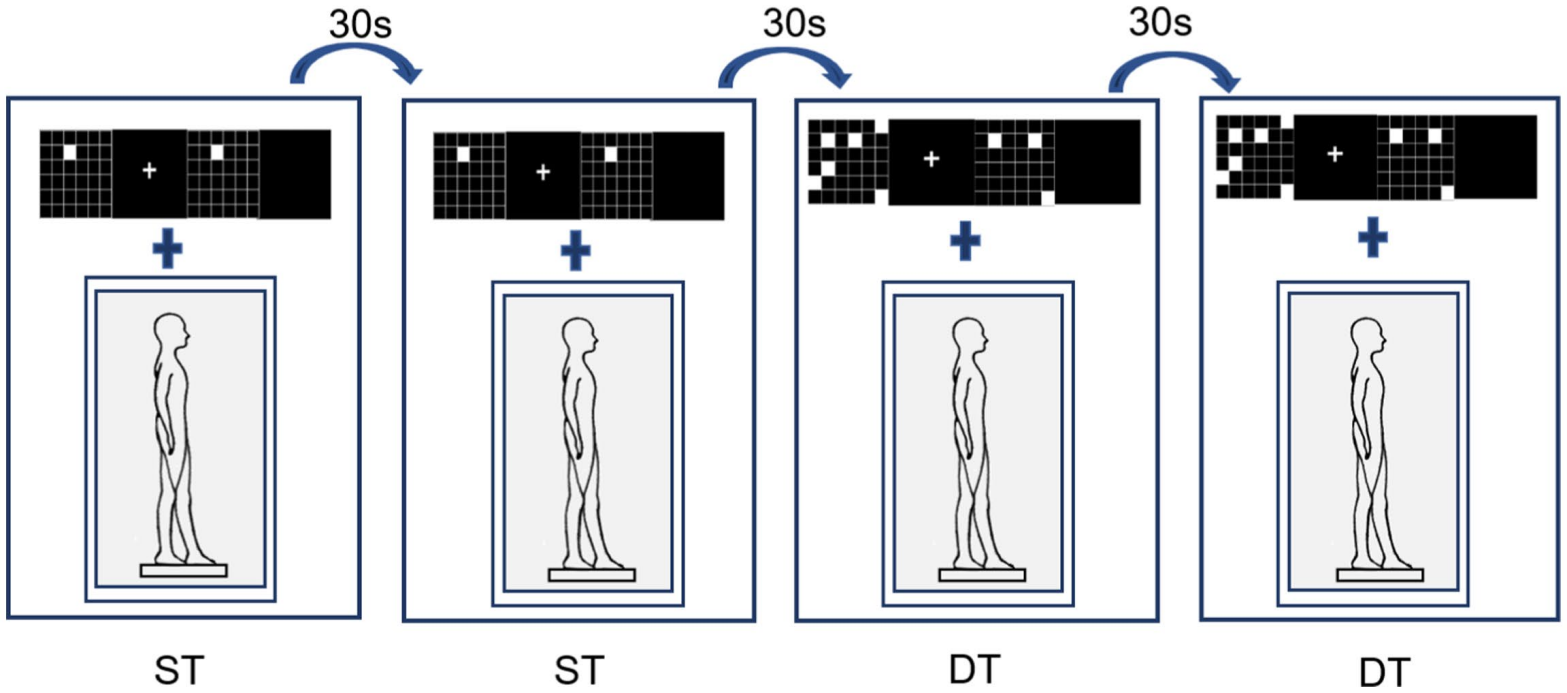
Single- and dual-task postural control assessment.

In the postural control single-task, participants were instructed to stand steadily and click on the mouse randomly when a white square appeared (cognitive control task). This cognitive control task (Figure 2, right) was used instead of standing without an additional task, to control articulatory movements that increase postural instability, thereby ensuring that the only additional component of the dual-task performance was working memory (Doumas et al., 2012). The dual-task assessment required participants to perform concurrently the visual-spatial match-to-sample task and tandem posture (Figure 3).

Postural control was measured by the sway velocity of center of pressure (COP) in the medial-lateral direction (V_ML_), the sway velocity of COP in the anterior-posterior direction (V_AP_), and the sway velocity of total trajectory oscillation (V_cop_). A larger speed of body sway is often interpreted as a phenomenon of less efficient postural control (Mitra et al., 2013).

### 2.4. rs-EEG recording

EEG was recorded with the Starstim^®^ (Neuroelectrics Inc., Barcelona, Spain) from 8 positions covering the left of the fronto-parietal cortex (F3, P3, Fz, CP2, F8, FC5, O2, AF3) with 3.14 cm^2^ Ag/AgCl electrodes and digitalized with 24-bit resolution at a sampling frequency of 500 samples/s. EEG data were referenced to the earlobe.

Pre-processing analysis of EEG data involved using EEGLAB 19.0 and the Matlab open-source toolbox (Mathworks, Natick, United States) (Battaglini et al., 2020). Offline, eyes-closed resting EEG data were band-pass filtered between 0.05 and 40 Hz (Butterworth filter, order = 2) (Battaglini et al., 2020). The continuous data were segmented into 1 s epochs to obtain 180 epochs. All epochs were visually inspected to remove data segments contaminated with muscular or ocular artifacts conditions. Independent component analysis (ICA) was used to correct electrode artifacts when required.

To detect the brain θ power spectrum following θ-tACS, the cleaned epochs were then used to extract the FFT spectrum. Finally, the individual power values in the frequency range of interest were averaged for each participant and separately for the pre- and post-stimulation sessions. Hereafter, we referred to “θ power” to indicate the average of power values extracted in the frequency range between 4 and 8 Hz.

To examine the effects of tACS on phase-locked activity, the phase locking value (PLV) between channel F3 and channel P3 of the θ band was computed. The instantaneous phases of each channel were estimated by applying a Hilbert transformation to the source signals filtered into a band between 4 Hz and 8 Hz. The PLV was then computed as a function of the instantaneous phase difference between channels F3 and P3 using the following equation (Lachaux et al., 1999):

$$PLV=\frac{1}{N}\left|\overset{N}{\underset{n=1}{\sum }}e^{i\left(\theta_{1}\left(n\right)-\theta_{2}\left(n\right)\right)}\right|$$

where *N* is the number of sampled time points, and θ_1_ and θ_2_ are the instantaneous phase values at time point *n*.

The PLV ranges between 0 and 1, where a value close to 0 indicates a random phase relationship and a value close to 1 indicates a fixed signal phase relationship. Finally, the PLV in θ band was averaged for each participant pre- and post-stimulation.

### 2.5. HD-tACS

HD-tACS was delivered via the StarStim8 device, which is a hybrid wireless neurostimulation system for simultaneous EEG and tACS, controlled by the Neuroelectrics Instrument Controller (NIC 2.0; http://www.neuroelectrics.com/products/software/nic2/). We used 7 PIS-TIM Ag/AgCl electrodes with a 1 cm radius for stimulation. The optimal electrode placement and current intensity were developed using Stimweaver^®^ optimization technique simulations (Miranda et al., 2013; Ruffini et al., 2014) based on the main stimulation target areas (the left dorsolateral prefrontal cortex and left posterior parietal cortex) of this study. The montage injects a total current of 2 mA with the target En-field was set to +0.25 V/m over each target region, and 0 V/m over the remaining regions.

**Figure 4:**
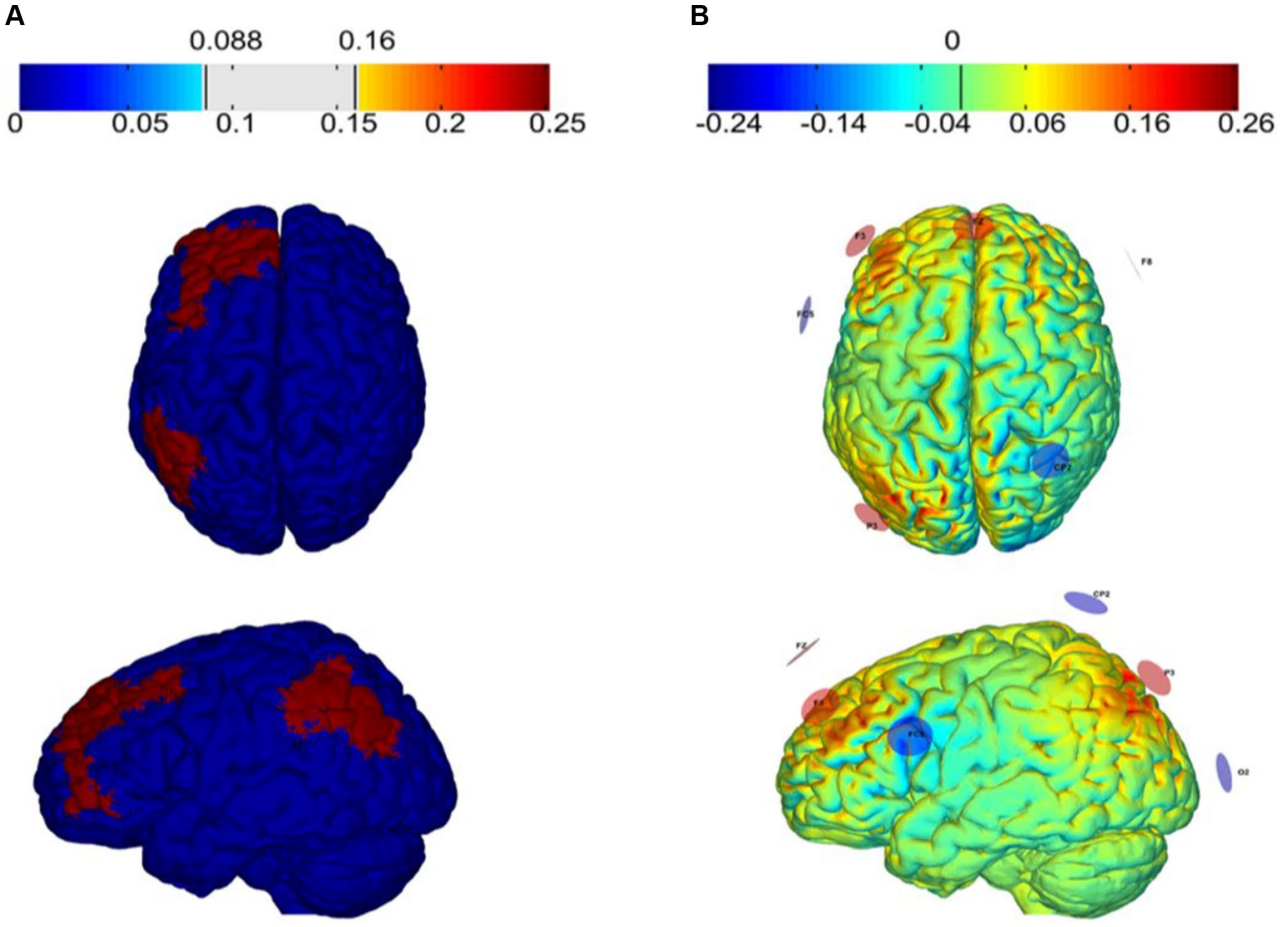
Electric field (V/m) of the target region **(A)** and tACS montage **(B)** The targets of tACS and modeling of the normal component of the electric field (En) over the cortex as induced by montages targeting the left dorsolateral prefrontal cortex (L-DLPFC) and the left posterior parietal cortex (PPC).

The tACS was delivered with the participant seated and resting for 20 min, including a 30 s fade in and a 30 s fade out. Aim to enhance the synchronization of θ oscillations between LDLPFC and LPPC (Polanía et al., 2012), the modeling of the tACS resulted in the placements of electrodes on the F3: 784 μA, P3: 831 μA, Fz: 384 μA, CP2: −402 μA, F8: −481 μA, FC5: −522 μA, O2: −594 μA of the 10–10 EEG placement system (Figure 4). The stimulation frequency was 6 Hz with a 0° relative phase. Sham tACS utilized the same montage yet the current was only applied for only 1 min (30 s fade in and 30 s fade out). All participants were asked to complete a questionnaire regarding the side effects and blinding efficacy of tACS at the end of each session (Fertonani et al., 2015).

### 2.6. Statistical analysis

Normality and homogeneity of variance were tested for all outcomes using the Shapiro–Wilk test and the Levene’s test. Independent samples *t*-tests were used to compare the difference in each outcome data before the stimulation intervention.

To investigate whether there was a dual-task interference effect, we used the one-way ANOVA to analyze behavioral data of single- and dual-task before the stimulation intervention at 2 visits (tACS or sham).

Primary analyses utilized two-way (stimulation condition × time) repeated-measures ANOVAs to examine the effects of tACS on working memory performance (ACC, RT, IES), θ power of rs-EEG (F3_PSD, P3_PSD) and PLV of θ band. Similar analyses were applied to the secondary outcomes including the performance of upright standing (V_ML_, V_AP_, and Vcop). Paired *t*-tests were used within groups to analyze the differences in each outcome before and after stimulation. ANOVA effect sizes are denoted using the bias *η*_p_^2^; Cohen’s *d* is used to show *t*-test effect sizes (where Cohen’s *d* < 0.19 is a weak effect, 0.20–0.49 is a low effect, 0.50–0.79 is a medium effect, and >0.80 is a high effect.)

To study how the tACS influences behavioral performance, the Pearson correlation analysis was used to examine the correlation between the change of resting-state EEG (∆F3_PSD, ∆P3_PSD, ∆PLV) and the change of behavioral outcomes (ΔACC, ΔRT, ΔIES, ΔV_ML_, ΔV_AP_, ΔV_cop_) after different stimulus conditions (post-pre).

Finally, the Mann–Whitney *U* test was used to examine the side effects of tACS, and the Fisher exact test was used to examine blinding efficacy.

The statistical software was SPSS 25.0 with significance *α* = 0.05.

## 3. Result

All 20 participants completed the study. Behavioral outcomes (ACC, RT, IES, V_ML_, V_AP_, Vcop), θ power of rs-EEG and PLV outcomes (F3_PSD, P3_PSD, PLV), separated by both stimulation conditions and time (pre-/post-stimulation), are presented in Table 2.

**Table 2:** The effects of tACS on working memory, postural control, and rs_EEG (mean ± SD).

Variables	tACS				Sham				*F*_1,19_	*p*
	Pre	Post	*p*	Cohen’s *d*	Pre	Post	*p*	Cohen’s *d*		
**Working memory**										
*ST*										
WM_ACC (%)	0.75 ± 0.1	0.73 ± 0.1	0.456	0.178	0.72 ± 0.1	0.75 ± 0.1	0.241	0.275	1.376	0.255
WM_RT (s)	0.86 ± 0.2	0.85 ± 0.2	0.494	0.163	0.83 ± 0.2	0.82 ± 0.2	0.197	0.188	0.019	0.893
WM_IES	1.18 ± 0.3	1.17 ± 0.3	0.890	0.032	1.16 ± 0.3	1.11 ± 0.3	0.159	0.329	0.483	0.496
*DT*										
WM_ACC (%)	0.75 ± 0.1	0.74 ± 0.1	0.822	0.050	0.73 ± 0.1	0.71 ± 0.1	0.502	0.170	0.069	0.795
WM_RT (s)	0.84 ± 0.2	0.79 ± 0.2	0.005^**^	0.742	0.82 ± 0.2	0.79 ± 0.2	0.216	0.300	0.417	0.526
WM_IES	1.14 ± 0.3	1.09 ± 0.2	0.356	0.215	1.10 ± 0.3	1.12 ± 0.2	0.765	0.073	0.588	0.453
**Postural control**										
*ST*										
V_ML_ (mm/s)	21.27 ± 4.5	20.83 ± 5.7	0.709	0.085	23.69 ± 4.8	24.78 ± 6.0	0.446	0.179	0.971	0.337
V_AP_ (mm/s)	19.32 ± 5.1	18.69 ± 7.0	0.606	0.117	20.07 ± 4.9	21.47 ± 7.3	0.16	0.336	3.602	0.074
Vcop (mm/s)	28.92 ± 5.9	28.26 ± 8.0	0.692	0.090	31.25 ± 6.0	32.98 ± 8.7	0.287	0.252	1.820	0.194
*DT*										
V_ML_ (mm/s)	19.97 ± 4.6	19.41 ± 4.1	0.320	0.229	23.31 ± 5.0	23.48 ± 4.1	0.846	0.046	0.344	0.565
V_AP_ (mm/s)	17.08 ± 4.4	16.67 ± 4.7	0.490	0.157	19.19 ± 5.3	19.02 ± 5.5	0.885	0.034	0.046	0.833
Vcop (mm/s)	26.40 ± 5.9	25.76 ± 5.5	0.383	0.200	30.20 ± 6.6	29.80 ± 4.8	0.735	0.079	0.038	0.848
*Rs_EEG*										
PSD_F3	−0.62 ± 2.7	−0.97 ± 2.1	0.411	0.188	−1.21 ± 1.7	−1.13 ± 1.9	0.827	0.049	1.163	0.294
PSD_P3	0.85 ± 2.5	0.74 ± 2.4	0.844	0.045	−0.11 ± 1.8	0.01 ± 1.7	0.605	0.115	0.179	0.677
PLV	0.31 ± 0.1	0.40 ± 0.1	0.010^*^	0.637	0.36 ± 0.1	0.38 ± 0.1	0.644	0.105	5.000	0.038^*^

ACC, accuracy; RT, rection time; IES, inverse efficiency scores; V_ML_, the velocity of medial-lateral sway trajectory; V_AP_, the velocity of anterior-posterior sway trajectory; V_cop_, the velocity of total sway trajectory oscillation; PSD_F3, θ power in channel F3; PSD_P3; PLV, phase locking value; θ power in channel P3.^*^Denote *p* < 0.05 and ^**^denote *p* < 0.01.

An independent t-test was used to compare the outcomes of pre-stimulation in the two stimulation conditions to assess consistency before stimulation, and the results showed no significant difference between the two groups (*p* > 0.05; Table 2).

### 3.1. Dual-task interference

When exploring the dual-task interference, we found no significant difference in working memory performance in either the upright posture or the seating conditions (*p* > 0.05) (see Table 3).

**Table 3:** Behavioral performance during single- and dual-task.

Variables (*n* = 20)	ST	DT	*F*_1,78_/*z*	*p*
*Working memory*				
WM_ACC	0.735 ± 0.08	0.735 ± 0.08	0.016	0.900
WM_RT	0.847 ± 0.18	0.828 ± 0.18	0.227	0.635
WM_IES	1.168 ± 0.30	1.123 ± 0.27	0.493	0.485
*Postural control*				
V_ML_	**21.85 ± 4.6**	21.56 ± 5.0	-2.000	0.045^*^
V_AP_	**18.25 ± 6.4**	**16.55 ± 5.9**	-2.900	0.004^**^
Vcop	30.05 ± 5.9	28.25 ± 6.4	1.644	0.204

ST_V_ML_, ST/DT_V_AP_ (highlighted in bold black) did not conform to a normal distribution, for which we used a non-parametric Wilcoxon signed-rank test for statistical analysis and, denoted by median ± quartiles. The rest of the data are represented as the mean ± SD, ^*^Denote *p* < 0.05 and ^**^ denote *p* < 0.01.

With respect to exploring the dual-task interference effects of postural control, as the data of single-task V_ML_ and single- and dual-task V_AP_ did not satisfy a normal distribution, a non-parametric correlation sample Wilcoxon signed-rank test was performed on this part of the results; we found that compared with those under the single-task, the V_ML_ (*z* = −2, *p* = 0.045) and V_AP_ (*z* = −2.9, *p* = 0.004) under dual-task conditions were significantly reduced by 0.4% and 8.0%, respectively (Table 3). These results indicate that there was less body sway during the concurrent execution of the working memory task; that is, working memory did not negatively interfere with upright postural control.

### 3.2. Effect of fronto-parietal θ-tACS on working memory

**Figure 5:**
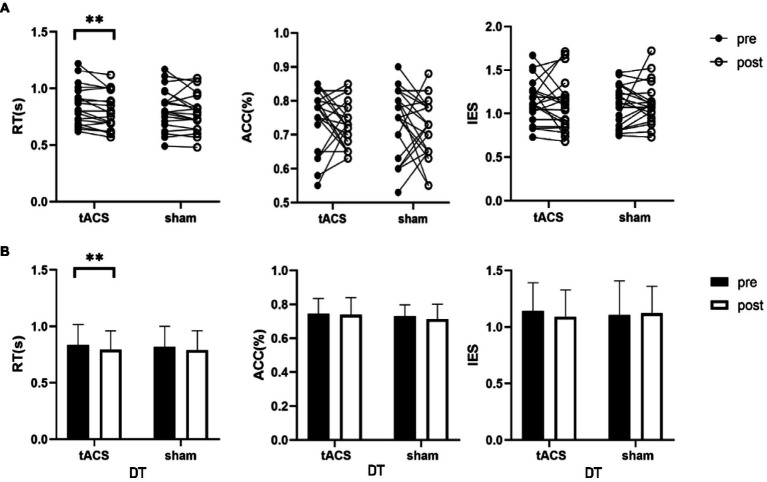
tACS effect on dual-task working memory. **(A)** The individual values of RT, ACC and IES, there was a significant decrease in RT after tACS. **(B)** Mean ± SD of RT, ACC and IES before and after tACS or sham stimulation. RT, reaction time; ACC, accuracy; IES, inverse efficiency score. Error bars represent SD, ^**^Denote *p* < 0.01.

No interaction was noted between the effects of the two groups before and after stimulation (stimulation condition × time) on single-task working memory ACC (*F*_1,19_ = 1.376, *p* = 0.225, *η*_p_^2^ = 0.068), RT (*F*_1,19_ = 0.019, *p* = 0.893, *η*_p_^2^ = 0.001) and IES (*F*_1,19_ = 0.483, *p* = 0.496, *η*_p_^2^ = 0.025) (Table 2). There were also no interactions for the effects on dual-task working memory ACC (*F*_1,19_ = 0.069, *p* = 0.795, *η*_p_^2^ = 0.004), RT (*F*_1,19_ = 0.417, *p* = 0.526, *η*_p_^2^ = 0.021), and IES (*F*_1,19_ = 0.588, *p* = 0.453, *η*_p_^2^ = 0.030) (Table 2). A paired *t*-test was used to analyze the within-group data and found a 5.3% significant decrease in working memory RT under dual-task conditions after tACS (*t* = −3.157, *p* = 0.005, Cohen’s *d* = 0.742, Figure 5). In contrast, no significant difference was observed in the RT after sham stimulation (*t* = −1.279, *p* = 0.216, Cohen’s *d* = 0.3). The ACC and IES during the dual task and the ACC, RT, and IES during the single task were unaffected by any stimulation (Table 2).

### 3.3. Effect of fronto-parietal θ-tACS on postural control

No significant interaction was observed for V_ML_ (*F*_1,19_ = 0.971, *p* = 0.337, *η*_p_^2^ = 0.051), V_AP_ (*F*_1,19_ = 3.602, *p* = 0.074, *η*_p_^2^ = 0.167) or Vcop (*F*_1,19_ = 1.820, *p* = 0.194, *η*_p_^2^ = 0.092) during single-task. There were also no interactions for V_ML_ (*F*_1,19_ = 0.344, *p* = 0.565, *η*_p_^2^ = 0.020), V_AP_ (*F*_1,19_ = 0.046, *p* = 0.833, *η*_p_^2^ = 0.002) or Vcop (*F*_1,19_ = 0.038, *p* = 0.848, *η*_p_^2^ = 0.148) during dual-task (Table 2). Paired t-tests revealed no significant differences in postural control performance before or after both stimulations (Table 2).

### 3.4. Effect of fronto-parietal θ-tACS on θ power and PLV

RM_ANOVA revealed no interaction for spectral power changes of θ bands in channel F3(*F*_1,19_ = 1.163, *p* = 0.294, *η*_p_^2^ = 0.58) or channel P3(*F*_1,19_ = 0.179, *p* = 0.677, *η*_p_^2^ = 0.009). Paired t-tests failed to identify regions with a significant power difference following any stimulation conditions (Table 2).

**Figure 6:**
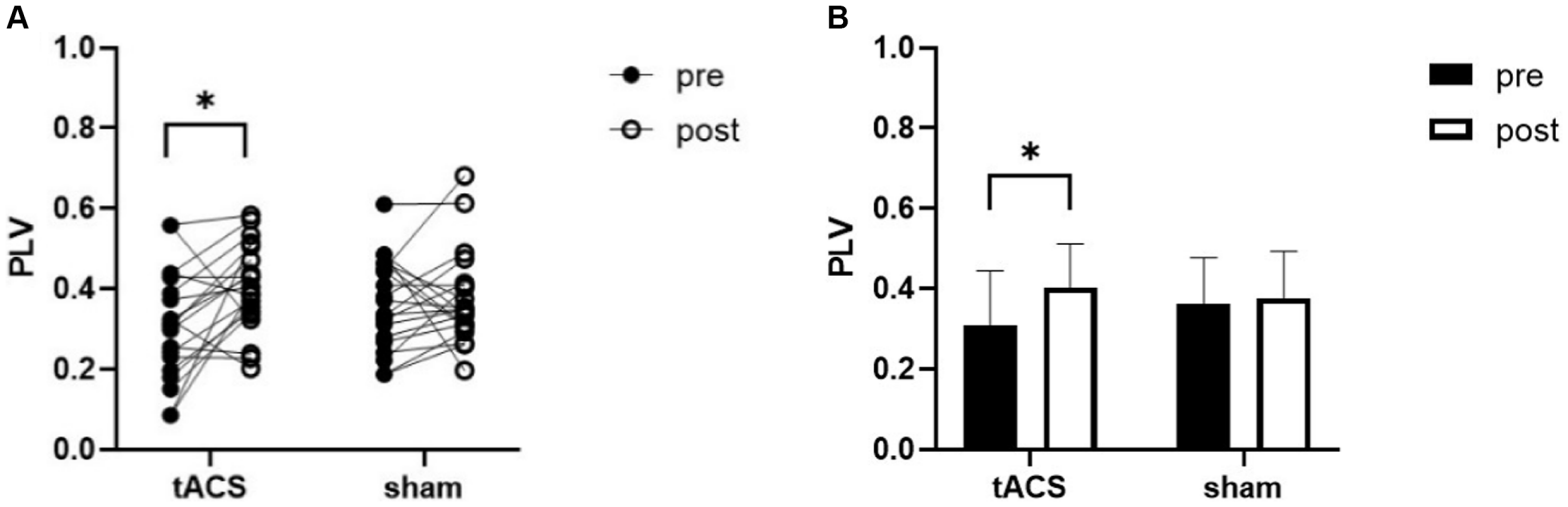
tACS effect on PLV of θ band. **(A)** the individual values of PLV, there was a significant increase after tACS. **(B)** Mean ± SD of PLV before and after tACS or sham stimulation. PLV, phase locking value. Error bars represent SD, ^*^denote *p* < 0.05.

RM_ANOVA revealed an interaction for PLV between channel F3 and channel P3 (*F*_1,19_ = 5, *p* = 0.038, *η*_p_^2^ = 0.208). Post-hoc testing indicated that the PLV increased after tACS compared with that in the sham group. A paired *t*-test was used to analyze within-group data and found a significant increase in PLV after tACS (*p* = 0.010, Cohen’s *d* = 0.637, Figure 6). However, no significant difference was observed after the sham stimulation (*p* = 0.644, Cohen’s *d* = 0.105).

### 3.5. Relationship between behavioral performance and rs-EEG

**Figure 7:**
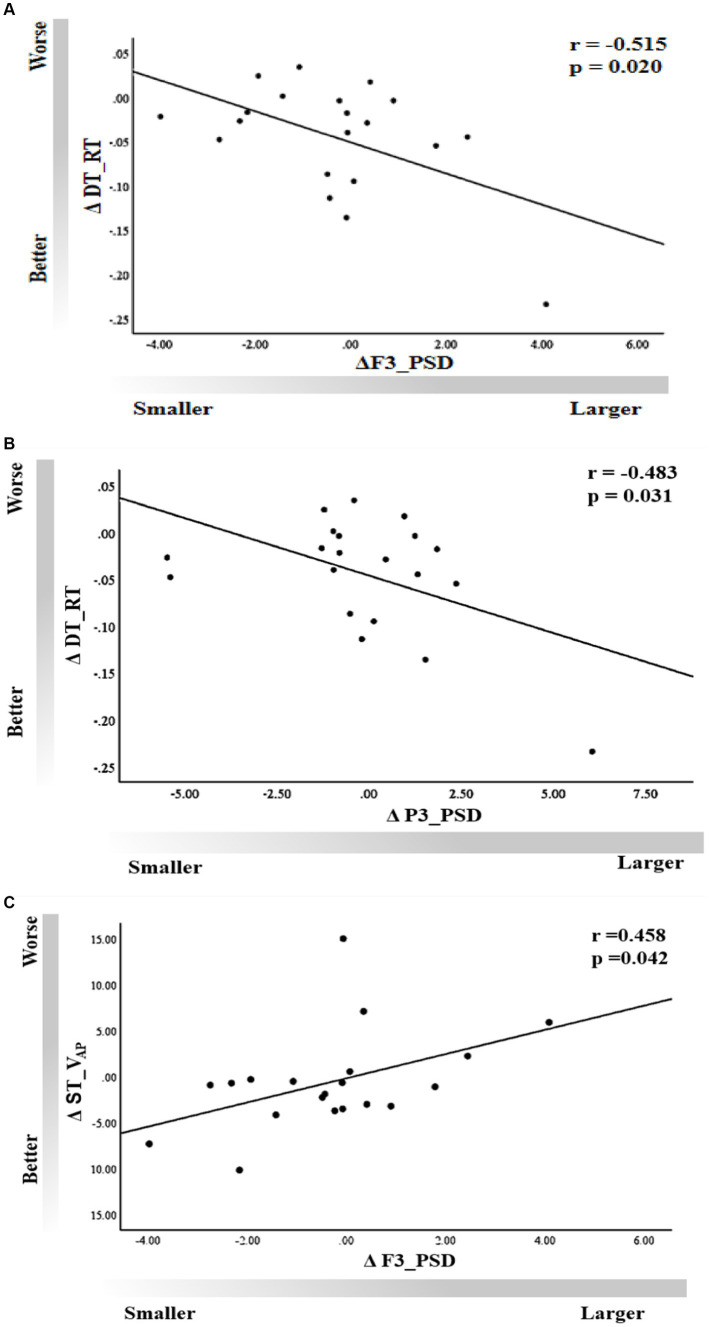
Correlation of dual-task working memory ΔRT with rs-EEG **(A)** ΔF3_PSD and **(B)** ΔP3_PSD. **(C)** Correlation of ΔV_AP_ with ΔF3_PSD under the single-task condition.

A moderate correlation was observed between ΔF3_PSD and ΔDT_RT (*r* = −0.515, *p* = 0.02), as well as between ΔP3_PSD and ΔDT_RT (*r* = −0.483, *p* = 0.031) in the tACS session (Figures 7A,B), indicating that improvements in working memory performance corresponded to an increase in the power spectra of channels F3 and P3. We also found a significant correlation between ΔV_AP_ and ΔF3_PSD in the single-task condition (*r* = 0.458, *p* = 0.042); that is, an increase in body sway velocity in the anteroposterior direction was significantly associated with an increase in power in the band of the F3 channel (Figure 7C). However, no significant associations were observed for other behavioral outcomes (*p* > 0.05).

A moderate correlation was observed between ∆PLV and ∆ST_RT (*r* = 0.455, *p* = 0.02) in the tACS session (Supplementary Table S1). No significant associations were observed for other behavioral outcomes (*p* > 0.05).

### 3.6. Side effects and blinding efficacy

Eight adverse events (itching, tingling, burning sensation, warmth, fatigue, metallic/iron taste, phosphene, and others) with five severity levels (none, mild, moderate, considerable, and strong) were reported in the side-effect questionnaire.

None of the participants reported serious or severe adverse events associated with tACS stimulation. Only three participants reported considerable itching, and one reported considerable tingling in the tACS group. The Mann–Whitney *U* test showed no significant difference in side effects between the two groups (*p* > 0.05) (Supplementary Table S2).

For blinding efficacy analysis, we only included guesses from the first visit because participants’ guesses on subsequent visits may have been influenced by subjective guesses from the first visit. Fisher’s exact test revealed no significant difference in the blinding efficacy between the two stimulation conditions (*p* = 0.103) (see Table 4).

**Table 4:** Blinding efficacy of tACS and sham stimulation within first visit.

	tACS (*n* = 10)	Sham (*n* = 10)	*p*
Accuracy	8 (80%)	3 (30%)	
Inaccuracy	1 (10%)	5 (50%)	
Uncertainty	1 (10%)	2 (20%)	
			0.103

## 4. Discussion

This study investigated the immediate effects of fronto-parietal θ-tACS on a dual-task that measured working memory and postural control. The results showed that tACS had positive effects on working memory while standing upright. The improvement in working memory performance was associated with increased EEG power, indicating that changes in behavior could result from the modulation of endogenous neural processes. Phase synchronization analysis revealed modulations in the θ band between F3 and P3 after tACS. However, there were no observable differences in the EEG power or upright postural control performance among the stimulus conditions. These results suggest that a single session of tACS may have limited effects on the working memory and postural control dual tasks.

### 4.1. Working memory

Neuropsychological studies have demonstrated that the frontalparietal network and brain region θ phase synchronization play an important role in the processing of WM. The tACS, as a tool for brain oscillatory modulation, can effectively elicit working memory-related neural oscillatory activity and improve WM performance (Polanía et al., 2012; Violante et al., 2017). In the present study, our results suggest that the WM reaction significantly improved after tACS. However, of note, the improvement in working memory in this study only occurred in conditions where upright postural control was performed (dual task). In contrast, WM performance in the seated position (i.e., single task) showed no significant change. This is not consistent with hypothesis 1, possibly because the demanding condition of limited resources under the dual-task condition is more sensitive to weak changes in the cognitive performance of healthy subjects. This may be due to differences in the brain mechanisms between single- and dual-task WM (Ozdemir et al., 2016). Our tACS montage, an HD-tACS with higher spatial accuracy than the traditional sponge tES montage, may modulate the brain regions of dual-task WM (Alam et al., 2016).

### 4.2. Postural control

Contrary to our initial experimental hypothesis, the current study did not show significant differences in postural control performance between the two stimulation conditions. Moreover, postural control performance did not deteriorate in the dual-task condition, but instead appeared to improve while simultaneously performing WM, which was also unexpected. We speculate that this might be due to the attentional focus shift, namely the dual-task performance in the present study, which increases the automatic processing of posture (McNevin and Wulf, 2002; Riley et al., 2003) and decreases body weight sway by shifting the focus of attention from standing performance (internal focus) to the execution of a working memory task (external focus) (Wulf et al., 2001). However, redundant eye movements may also have an impact when performing visual cognitive tasks (Bonnet and Baudry, 2016; Bonnet et al., 2021). Previous reports have shown a synergistic relationship between the postural and visual systems, with the central neural system possibly guiding a more stable postural state to complete visual-cognitive tasks (Bonnet and Baudry, 2016; Bonnet et al., 2021).

Based on these findings, it is challenging to further investigate whether tACS influences postural control by improving working memory. As standing performance was already better in the dual-task situation (compared to single-task postural control performance), even if tACS had a positive effect on postural control during a working memory task, it may not have been well assessed. Future studies could select more difficult or real-life postural control tasks, such as throwing (Zhuang, 2021) or dynamic postural control tasks (standing on a translation platform, walking, and obstacle crossing) (Bogost et al., 2016; Lin and Lin, 2016; Ozdemir et al., 2016; Nóbrega-Sousa et al., 2020). In addition, as biological aging and age-related conditions appear, our cognitive function and brain mechanisms become increasingly crucial in preserving our ability to maintain balance (Manor et al., 2010; Manor and Lipsitz, 2013); future work could focus on older adults (Rizzato et al., 2021) to prevent ceiling effects.

### 4.3. Correlation analysis of EEG and behavioral data

Correlation analyses revealed a relationship between the change in EEG θ power and WM response time, i.e., an increase in θ power after tACS corresponded to an accelerated response to a WM task in the present study, which in part supports a link between the modulation of endogenous neural processes and changes in behavior. Available evidence suggests that the primary mechanisms by which tACS modulates the brain are entrainment of endogenous rhythms at the frequency of stimulation (Zaehle et al., 2010; Herrmann et al., 2013) and induction of synaptic changes via spike-timing-dependent plasticity (Zaehle et al., 2010; Vossen et al., 2015). The fronto-parietal in-phase θ-tACS in the present study may modulate endogenous θ oscillations in the brain with exogenous θ oscillations, allowing behaviorally relevant neural oscillatory networks (i.e., fronto-parietal θ phase synchronization) to be driven synchronously (Reinhart and Nguyen, 2019).

### 4.4. Frequency-specific EEG aftereffects of tACS

PLV analyses showed increased θ phase synchronization between frontal and parietal brain regions after tACS, as opposed to sham. The PLV is an indicator of phase synchronization across cortical regions, and previous studies have shown that phase synchronization can alter spike-time-related plasticity (Gregoriou et al., 2009; Wang, 2010). We speculate that external modulation of θ phase synchronization improves WM possibly due to neuroplasticity changes in functional connectivity. Intervention in the temporal synchronization patterns of large-scale human brain activity via tACS has the potential to enhance the postsynaptic effects of spiking impulses in one region on another, ultimately improving neural communication related to working memory capacity (Reinhart and Nguyen, 2019). However, contrary to our hypothesis, no significant changes in θ power values were observed after tACS in the present experiment. Studies that have investigated the effect of tACS on brain oscillatory power reported inconsistent results. Some studies reported a decrease in endogenous power values after tACS (Pahor and Jaušovec, 2017), while others found an increase in power values. Additionally, some studies found no change (Wischnewski et al., 2016) possible reason for the mixed evidence above is due to the heterogeneity of the variables (variables vary in terms of amplitude, power, and relative ratios) (Wischnewski et al., 2016; Kleinert et al., 2017; Pahor and Jaušovec, 2017; Battaglini et al., 2020).

### 4.5. Limitations and prospects

In the current study, the transcranial electrical stimulation (tES) protocol used a high-definition stimulation montage design (HD-tACS). To date, few studies have used HD-tACS (Reinhart and Nguyen, 2019; Klírová et al., 2021), rendering the application of HD-tACS in this study a possible limitation. HD-tES has the advantage of higher spatial accuracy than the traditional sponge tES montage, while the latter produces more diffuse currents throughout the brain (Datta et al., 2009; Ruffini et al., 2014; Alam et al., 2016). However, the more concentrated the current pattern produced by HD-tES, the less modulation of relatively distant brain regions of the target function may be achieved, ultimately resulting in weakening of the modulation effect (Hill et al., 2019). Overall, the effects of focused current patterns produced by HD-tACS must be investigated in detail. Furthermore, the physiology of a participant’s head is quite variable (Truong et al., 2013; Opitz et al., 2015) and the method of locating the stimulation target area using the same EEG cap may ultimately result in the actual stimulation site deviating from the ideal stimulation target area. Even more, high definition montage designs can amplify this limitation (Mikkonen et al., 2020). To this end, magnetic resonance imaging can be used to determine individual target areas (Datta et al., 2012; Mikkonen et al., 2020; Klírová et al., 2021).

## 5. Conclusion

Fronto-parietal HD-tACS at 6hz improved working memory performance in healthy young adults in dual-task situations. The improvement in working memory performance was also associated with an increase in EEG θ power. Furthermore, tACS interferes with the temporal synchronization patterns of large-scale human brain activity and improves neural communication associated with WM. However, this protocol did not affect upright postural control. In summary, fronto-parietal θ HD-tACS has the potential of being a neuromodulatory tool for improving working memory performance in dual-task situations, but its effect on the modulation of concurrently performed postural control tasks requires further investigation.

## Data availability statement

The original contributions presented in the study are included in the article/Supplementary material, further inquiries can be directed to the corresponding authors.

## Ethics statement

The studies involving humans were approved by the institutional review board of the Shanghai University of Sports (102772020RT109). The studies were conducted in accordance with the local legislation and institutional requirements. The participants provided their written informed consent to participate in this study.

## Author contributions

YX: Formal analysis, Methodology, Writing – original draft, Conceptualization. JZ: Formal analysis, Writing – original draft. RZ: Conceptualization, Investigation, Methodology, Writing – review & editing. YL: Funding acquisition, Project administration, Supervision, Writing – review & editing. JL: Conceptualization, Supervision, Writing – review & editing. LH: Conceptualization, Project administration, Supervision, Writing – review & editing.

## Figures and Tables

**Table:** 

ACC	Accuracy
COP	Center of pressure
DT	Dual-task
EEG	Electroencephalography
HD-tACS	High-definition transcranial alternating current stimulation
ICA	Independent component analysis
IES	Inverse efficiency score
lDLPFC	Left dorsolateral prefrontal cortex
PLV	Phase locking value
PPC	Posterior parietal cortex
PSD	Power spectral density
RM-ANOVA	Repeated measures ANOVA
rs-EEG	Resting-state electroencephalography
RT	Reaction time
ST	Single-task
tACS	Transcranial alternating current stimulation
tES	Transcranial electrical stimulation
WM	Working memory
