# Supplementary material for: Fronto-parietal theta high-definition transcranial alternating current stimulation may modulate working memory under postural control conditions in young healthy adults
Source: Front Hum Neurosci. 2023 Nov 6;17:1265600. doi: 10.3389/fnhum.2023.1265600 (PMC10666918; doi:10.3389/fnhum.2023.1265600)
Supplement: Supplementary file 1 [file Table_1.docx]

**Supplementary Table 1.** Relationship between behavioural performance and PLV

| **Variable (post-pre)** | **tACS** | |  | **sham** | |
| --- | --- | --- | --- | --- | --- |
|  | ***r*** | ***p*** |  | ***r*** | ***p*** |
| **Working memory ＆ PLV** |  |  |  |  |  |
| **ST** |  |  |  |  |  |
| ΔACC ＆ ΔPLV | -0.13 | 0.585 |  | 0.067 | 0.78 |
| ΔRT ＆ ΔPLV | 0.455 | 0.044***** |  | -0.026 | 0.914 |
| ΔIES＆ ΔPLV | 0.13 | 0.351 |  | -0.156 | 0.511 |
| **DT** |  |  |  |  |  |
| ΔACC ＆ ΔPLV | -0.167 | 0.481 |  | -0.15 | 0.529 |
| ΔRT ＆ ΔPLV | 0.332 | 0.153 |  | -0.213 | 0.366 |
| ΔIES＆ ΔPLV | 0.242 | 0.305 |  | 0.01 | 0.967 |
| **postural control ＆ PLV** |  |  |  |  |  |
| **ST** |  |  |  |  |  |
| V_ML_＆ΔPLV | 0.099 | 0.679 |  | 0.375 | 0.114 |
| V_AP_ ＆ΔPLV | 0.012 | 0.958 |  | 0.128 | 0.602 |
| Vcop ＆ ΔPLV | 0.064 | 0.789 |  | 0.298 | 0.215 |
| **DT** |  |  |  |  |  |
| V_ML_ ＆ ΔPLV | 0.144 | 0.544 |  | 0.164 | 0.502 |
| V_AP_ ＆ ΔPLV | -0.278 | 0.235 |  | 0.03 | 0.903 |
| Vcop ＆ ΔPLV | -0.083 | 0.727 |  | 0.028 | 0.909 |

*** denote p < 0.05
