# Supplementary material for: Fronto-parietal theta high-definition transcranial alternating current stimulation may modulate working memory under postural control conditions in young healthy adults
Source: Front Hum Neurosci. 2023 Nov 6;17:1265600. doi: 10.3389/fnhum.2023.1265600 (PMC10666918; doi:10.3389/fnhum.2023.1265600)
Supplement: Supplementary file 2 [file Table_2.docx]

**Supplementary Table 2. The report of side effects in tACS and sham stimulation (%)**

| **Side effects** |  | **The degree of intensity** | | | | | |
| --- | --- | --- | --- | --- | --- | --- | --- |
|  |  | None | mild | Moderate | Considerate | Severe | *p* |
| Itchiness |  |  |  |  |  |  |  |
|  | tACS | 25.0% (5) | 45.0% (9) | 15.0% (3) | 15.0% (3) | 0 | 0.468 |
|  | sham | 30.0% (6) | 50.0% (10) | 15.0% (3) | 5.0% (1) | 0 |  |
| tingling |  |  |  |  |  |  |  |
|  | tACS | 55.0% (11) | 40.0% (8) | 0 | 5.0% (1) | 0 | 0.291 |
|  | sham | 70.0% (14) | 30.0% (6) | 0 | 0 | 0 |  |
| Burning |  |  |  |  |  |  |  |
|  | tACS | 90.0% (18) | 10.0% (2) | 0 | 0 | 0 | 0.103 |
|  | sham | 70.0% (14) | 20.0% (4) | 5.0% (1) | 5.0% (1) | 0 |  |
| Warmth/heat |  |  |  |  |  |  |  |
|  | tACS | 65.0% (13) | 30.0% (6) | 5.0% (1) | 0 | 0 | 0.667 |
|  | sham | 70.0% (14) | 30.0% (6) | 0 | 0 | 0 |  |
| Metallic/iron taste |  |  |  |  |  |  |  |
|  | tACS | 95.0% (19) | 5.0% (1) | 0 | 0 | 0 | 0.553 |
|  | sham | 90.0% (18) | 10.0% (2) | 0 | 0 | 0 |  |
| Fatigue |  |  |  |  |  |  |  |
|  | tACS | 80.0% (16) | 15.0% (3) | 5.0% (1) | 0 | 0 | 0.359 |
|  | sham | 90.0% (18) | 10.0% (2) | 0 | 0 | 0 |  |
| Phosphene |  |  |  |  |  |  |  |
|  | tACS | 95.0% (19) | 5.0% (1) | 0 | 0 | 0 | 0.553 |
|  | sham | 90.0% (18) | 10.0% (2) | 0 | 0 | 0 |  |
| Other |  |  |  |  |  |  |  |
|  | tACS | 95.0% (19) | 5.0% (1) | 0 | 0 | 0 | 0.534 |
|  | sham | 90.0% (18) | 5.0% (1) | 5.0% (1) | 0 | 0 |  |
